# The impact of COVID-19 pandemic on mental and physical wellbeing in women with fibromyalgia: a longitudinal mixed-methods study

**DOI:** 10.1186/s12905-022-01840-9

**Published:** 2022-06-30

**Authors:** Asimina Lazaridou, Myrella Paschali, Eric S. Vilsmark, Timothy Wilkins, Vitaly Napadow, Robert Edwards

**Affiliations:** 1 Department of Anesthesiology, Harvard Medical School, Brigham & Women’s Hospital, Pain Management Center, 850 Boylston St, Suite 302, Chestnut Hill, MA 02467 USA; 2 MGH/MIT/HMS Athinoula A. Martinos Center for Biomedical Imaging, Charlestown, MA USA; 3 Fielding Graduate University, Santa Barbara, CA USA

**Keywords:** COVID-19, Pandemic, Fibromyalgia, Impact, Mental health, Catastrophizing

## Abstract

**Objective:**

The COVID-19 pandemic has strongly influenced psychological and physical health worldwide. The aim of this study was to examine the impact of the pandemic on women with fibromyalgia.

**Methods:**

This mixed methods pilot study explored measures of pain severity and interference, as well as pain catastrophizing and level of fibromyalgia impact among women with fibromyalgia before and during the COVID-19 pandemic in the USA. Fibromyalgia patients completed demographic, pain-related, and other validated psychosocial questionnaires prior to the onset of the COVID-19 pandemic, and then were re-assessed with those questionnaires, as well as a pandemic-related questionnaire assessing the impact of the pandemic on the patients’ life, during the pandemic.

**Results:**

When comparing data reported before the pandemic to data collected 3–6 months into the pandemic, women with fibromyalgia reported a general worsening of their pain and pain-related symptoms. During the pandemic, pain catastrophizing (*p* ≤ 0.05) and fibromyalgia impact (*p* ≤ 0.05) increased significantly compared to before the pandemic. The increase in pain catastrophizing scores was highly correlated with the impact of the pandemic on the participants’ ability to cope with pain and on their mental health. Qualitative analysis corroborated the significant impact of the pandemic on patients’ mental health, with the vast majority reporting a worsening of their mood. Other impacted domains included anxiety, level of activity and sleep.

**Conclusions:**

Collectively, the pandemic appears to have produced a substantive worsening of pain-related symptomatology among women with fibromyalgia, which should be addressed by targeted interventions.

**Supplementary Information:**

The online version contains supplementary material available at 10.1186/s12905-022-01840-9.

## Introduction

On 12 January 2020, the World Health Organization (WHO) confirmed that a novel coronavirus was the cause of a respiratory illness in a cluster of people in Wuhan, China, which was reported to the WHO in December 2019. The outbreak of the new SARS-CoV-2 (COVID-19) became a significant public health crisis that strongly influenced psychological and physical health worldwide. In the USA, a variety of isolation-based measures were implemented: these included recommendations to engage in social distancing, avoiding group gatherings, using remote audio and video interactions as a substitute for in-person contact, state and local lockdowns, and periods of quarantine. Reports concerning the psychological effects of social distancing and self-isolation during past epidemics and pandemics confirmed that quarantined people had higher risk of mental health symptoms including psychological distress, sleep disturbance and depression [1], and similar impacts of COVID-19 measures are also being reported [2].

Patients with chronic pain may have faced an increased burden during the pandemic due to limited access to healthcare and resources. Chronic pain is a common condition worldwide and is frequently associated with decreased health-related quality of life and high levels of negative affect [3]. Fibromyalgia (FM) is a musculoskeletal condition characterized by widespread persistent pain complaints, high levels of disability, and impairment in multiple domains of functioning such as emotional, work and social life (4–6). FM patients are advised to follow interdisciplinary treatment plans for pain management [6]. Due to the pandemic-related lockdown measures, many patients with chronic pain were forced to change their clinical management: scheduled visits were cancelled, in-person interventions (e.g. injections, physical therapy, acupuncture) were delayed, and therapeutic sessions were postponed, while telemedicine was rapidly introduced into clinical practice. As there is evidence of a high prevalence of psychiatric comorbidities in FM, especially depression, anxiety and post-traumatic stress disorder [7], these patients may be at elevated risk of experiencing a worsening of psychiatric symptomatology. Past studies have demonstrated a strong correlation between trauma, dissociation, and FM symptomatology [8, 9]. In addition, early traumatic experiences seems to be a risk factors for developing widespread pain in adulthood including FM symptoms by excessively activating stress responses during a critical period of development through hyperalgesic priming [10].Hence, FM patients may be particularly vulnerable to pandemic-related increase of depression and anxiety and possibly worsening of their clinical pain [11–13]. However, as the COVID-19 pandemic upended everyday life, many online surveys demonstrated that negative affect also increased among the general population [14, 15]. Additionally, Salaffi et al. demonstrated that contracting the virus led to a worsening of the disease impact on sleep quality, energy levels, pain levels, and stiffness [16]. A previous ambispective study reported an increase of pain and FM impact five months after the pandemic [17]. Our study aimed at evaluating whether and to what extent the changes occurring during the COVID-19 health emergency affected FM patients’ pain, mental health, ability to cope, and physical functioning utilizing qualitative and quantitative measures.

## Methods

### Study design

Between July 1st and November 30th 2020, 100 female FM patients who participated in a prior trial of a psychosocial intervention for chronic pain were contacted and invited to participate in a study assessing the effects of the COVID-19 pandemic on women with chronic pain. Out of the 100 contacted, only 38 agreed to participate in this study.

### Procedures

The demographic data, clinical characteristics and outcome data were obtained from the patients' electronic records before the pandemic (and study intervention) as well as through surveys collected through REDcap during the pandemic. Data were collected as comprehensively as possible through a combination of quantitative and qualitative data. Study procedures received approval by the Institutional Review Board (IRB) of Brigham & Women’s Hospital (Boston, MA, USA). We did follow the STROBE checklist for cross sectional studies.

### Eligibility criteria

Inclusion Criteria for FM patients:

1. Aged 18–65
2. Female
3. Have a clinical diagnosis of fibromyalgia and meet the Wolfe et al. 2011 research criteria for fibromyalgia
4. Baseline pain intensity of at least 4/10 on average and pain report for at least 50% of days
5. Able to provide written consent

Exclusion Criteria for FM:

1. Comorbid acute pain condition
2. Comorbid chronic pain condition that is rated by the subject as more painful than fibromyalgia
3. Current use of prescription stimulant medications (e.g., modafinil)
4. Routine use of substances of abuse

### Clinical measures

#### Clinical pain

To measure pain, we used the Brief Pain Inventory (BPI), a 15-item measure, that consists of two multi-item sub-scales that measure pain intensity and pain interference with daily activities. The BPI is well-validated in chronic pain and is frequently recommended as an outcome measure of pain severity and pain interference [18].

#### Pain catastrophizing

The Pain Catastrophizing Scale (PCS) is a widely used, self-report measure of catastrophic thinking associated with pain. The PCS consists of three subscales: rumination, magnification, and helplessness. The PCS is well-validated in chronic pain and has good psychometric properties in pain patients and controls [19].

#### Fibromyalgia impact

The Fibromyalgia Impact Questionnaire-Revised (FIQR) is 21-question measure with an 11-point numeric rating scale (NRS) of 0 to 10, with 10 being “worst”. The questionnaire is divided into three domains assessing: (a) “function”, (b) “overall impact” and the overall impact symptom severity, and (c) “symptoms”. All questions relate to the course of the past 7 days [20].

#### COVID-questionnaire

In order to assess the impact of COVID-19 on patients’ lives, we developed a questionnaire that included structured and open-ended questions (e.g., “What coping strategies have you been using to make daily life easier while staying at home/working from home /resuming your work responsibilities during the pandemic?”). The questionnaire consisted of two parts. The first included questions assessing the overall impact of the pandemic on the life of the patient (diagnosis of the patient or a relative with COVID-19, impact on medical care, utilization of virtual therapies, impact on ability to meet daily needs). The second part was focused on assessing the impact of the pandemic on FM symptoms (i.e. pain, fatigue and other symptoms), ability to cope with pain, and mental health.

### Data analysis

SPSS (v26, IBM Corp., Armonk, NY, USA) was used for all data analyses. The Wilcoxon signed-rank test (2-tailed, alpha = 0.05) was used to compare pre-Covid measurements (i.e., these “baseline” measurements were collected following the psychosocial intervention but prior to the onset of the pandemic) with those collected during the Covid-19 pandemic. The pre-post change score (ΔS) was calculated for each clinical measure. We calculated Spearman’s correlations to explore the relationships between these pre-post change scores and pandemic-related outcome variables. For the open-ended qualitative data, thematic analysis was chosen due to its flexibility, in which investigators identify relevant, frequently occurring information that is then categorized in emergent themes [21]. Two independent study team members individually assessed the responses to the open-ended questions and identified themes. For example, several comments referred to participants’ concerns related to employment and financial difficulties. These comments were categorized under the theme “Job loss”. The four criteria of credibility, dependability, confirmability, and transferability were used to refine themes until thematic saturation was achieved [22]. Points of agreement, disagreement, and universality were considered, and major themes were finalized.

### Power analysis

An a priori power analysis was conducted using G*Power version 3.1.9.7 to determine the minimum sample size required to test the study hypothesis. Results indicated the required sample size to achieve 80% power for detecting a medium effect, at a significance criterion of α = 0.05, was N = 28.

## Results

A total of 38 FM subjects agreed to participate in this pandemic-related questionnaire and complete the BPI, PCS and FIQR and the pandemic-related questionnaire in the months between July and November 2020. The sociodemographic characteristics of the enrolled patients, as measured before the pandemic, are presented in Table 1.

**Table 1:** Sociodemographic variables (before the pandemic)

Sociodemographic variables	*N* = *38*
Age (mean ± SD)	43.13 ± 11.38
Caucasian	81.6%
African American	7.9%
Hispanic/Latino	2.6%
Other	7.9%
Employed	50%
Married	39.5%
Living alone	15.8%
Education Level (college/masters/doctorate degree)	65.8%
Annual Income (above $45,000)	60.5%

SD: standard deviation

### Quantitative results

#### Changes in pain and pain-related outcomes associated with the pandemic

**Fig. 1:**
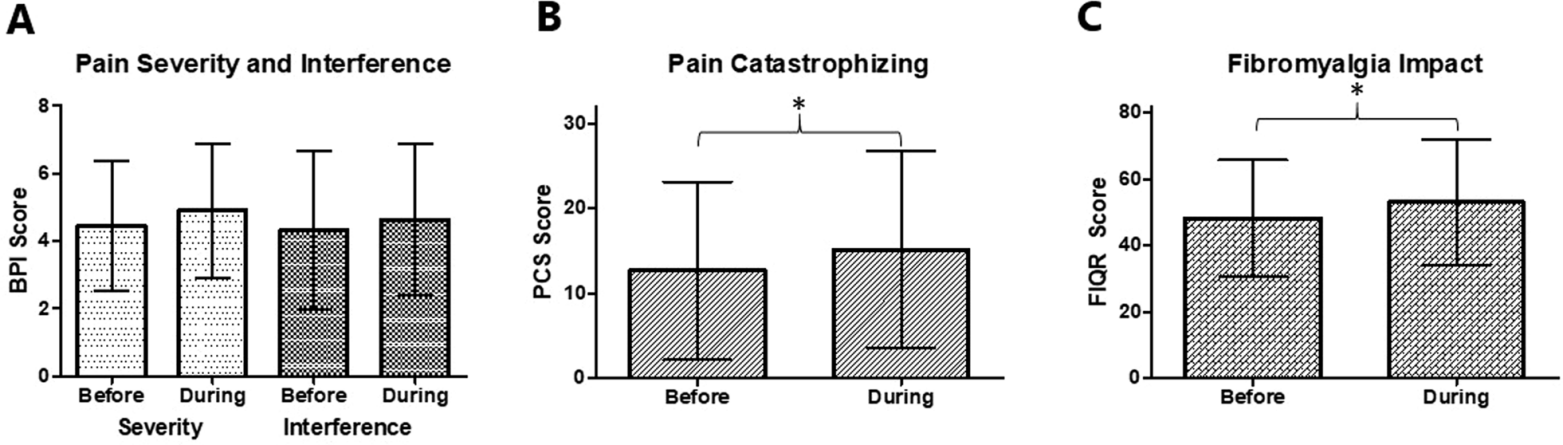
Pain intensity and interference (**A**), pain catastrophizing (**B**) and fibromyalgia impact (**C**) before and during the COVID-19 pandemic (mean scores ± SD). BPI: Brief Pain Inventory; PCS: Pain Catastrophizing Scale; FIQR: Fibromyalgia Impact Questionnaire Revised; *: *p* < 0.05

Thirty-five participants provided complete data for BPI, PCS and FIQR. Mean scores of all the scales are presented in Table 2. During the pandemic, pain catastrophizing (*p* ≤ 0.05) and FM impact (*p* ≤ 0.05) increased significantly compared to before the pandemic. There was no significant change in pain intensity and interference scores (Fig. 1). The inter-correlations of the difference scores (ΔS) for BPI, PCS and FIQR, as well as their correlations with pandemic-related outcome variables are shown on Table 3. PCS and FIQR change scores were shown to be correlated with the impact of the pandemic on the ability to cope with pain and participants’ mental health. The association between the change in PCS and the impact of the pandemic on enjoyment of life was nearly significant (*p* = 0.06).

**Table 2:** Clinical characteristics of participants before and during the COVID-19 pandemic

	Before the pandemic (n = 35)	During the pandemic (n = 35)	*p* value
BPI Severity	4.44 ± 1.93	4.91 ± 1.99	0.09
BPI Interference	4.33 ± 2.34	4.64 ± 2.23	0.48
PCS	12.63 ± 10.50	15.14 ± 11.57	≤ 0.05*
FIQR	48.24 ± 17.52	53.04 ± 18.99	≤ 0.05*

BPI: Brief Pain Inventory; PCS: Pain Catastrophizing; FIQR: Fibromyalgia Impact Questionnaire-Revised; PROMIS: Patient-Reported Outcomes Measurement Information System (T-Score)

**Table 3:** Correlations (Spearman’s) between pre-post change scores and pandemic-related outcome variables

	1	2	3	4	5	6	7	8	9
1. ΔS BPI Severity	1								
2. ΔS BPI Interference	0.59**	1			-				
3. ΔS PCS	0.41*	0.41*	1						
4. ΔS FIQR	0.54**	0.59**	0.60**	1					
5. Overall impact of pandemic on life	− 0.05	− 0.09	0.16	0.10	1				
6. Impact of pandemic on coping with pain	0.17	0.36*	0.50**	0.35*	0.41*	1			
7. Impact of pandemic enjoyment of life	− 0.02	0.21	0.31	0.19	0.40*	0.37*	1		
8. Impact of pandemic mental health	− 0.08	0.09	0.52**	0.33*	0.59**	0.56**	0.44**	1	
9. Impact of pandemic on medication use	0.05	− 0.21	− 0.23	− 0.05	0.03	− 0.17	− 0.28	− 0.21	1

^*^p < 0.05; **p < 0.01; ***p < 0.001

^**^Correlation is significant at the 0.01 level (two-tailed), ΔS: Delta change score; BPI: Brief Pain Inventory; PCS: Pain Catastrophizing; FIQR: Fibromyalgia Impact Questionnaire-Revised

#### General results of the pandemic in women with FM

All 38 participants answered the pandemic-related qualitative questions (see Additional file 1: Appendix). A vast majority of the participants (89%) reported that the COVID-19 pandemic has impacted their life. The overall impact of the pandemic was reported as negative by most participants. Specifically, 21% found the impact to be slightly negative, 39% somewhat negative, and 24% very negative. Only six participants reported to either have been formally diagnosed with COVID-19 or have had symptoms consistent with COVID-19 without a laboratory diagnosis. Most of the participants experienced worse access to medical care and did not feel that medical treatments or services provided via telehealth were sufficient to cover their pain-related needs (Table 4).

**Table 4:** Results of pandemic-related questionnaire: General Impact, Fibromyalgia Symptoms and Mental Health

n %		
Has experienced a negative impact on life overall	84.3	
Was diagnosed with COVID-19		
Confirmed with a laboratory test	5.3	
Developed symptoms consistent with COVID-19 but was never tested	10.5	
A member of their household was diagnosed with COVID-19		
Confirmed with a laboratory test	2.6	
Developed symptoms consistent with COVID-19 but was never tested	10.5	
Has experienced worse access in medical care since the pandemic	73.7	
Has received fibromyalgia-related medical treatment via telehealth	42.1	
Has felt that virtual therapies are sufficient for their pain-related needs	34.2	
Has experienced impacted ability to meet basic needs (e.g.housing, food)	63.1	
Pain has worsened	63.5	
Ability to cope with pain has worsened	66.2	
Pain medication intake changes		
Increased	21	
Decreased	10.5	
Fatigue has worsened	78.4	
Other fibromyalgia symptoms have worsened	65.8	
Mental well-being negatively impacted	86.8	
Enjoyment of life negatively impacted	73.7	
Mood has worsened	89.2	
Anxiety has increased	75.7	
Activity has decreased	68.3	
Sleep has worsened	76.3	

#### Fibromyalgia symptoms and mental health during the pandemic

The results of the second part of the pandemic-related questionnaire that was designed to capture FM and mental health symptoms are shown in Table 4.

Around two-thirds of the participants reported increase in their pain. Specifically, 32% felt that their pain became minimally worse, 21% much worse, and 10% very much worse. Interestingly, this worsening was not mirrored by the BPI results. Only a few participants (6%) found that their pain improved during the pandemic. Thirty-one percent of the participants stated that their pain remained unchanged. When patients were asked about the influence of the pandemic on their pain medication intake, the most common response was “no change” (68%). Most of the surveyed patients reported worsening of their fatigue and other FM symptoms. Additionally, the participants’ responses revealed a significant impact of the pandemic on their mental health, with the vast majority reporting a worsening of their mood. Other domains included worsening in their anxiety, level of activity and sleep.

#### Qualitative results using open-ended questions

Three themes were identified when reviewing the respondent’s answers related to how they were affected by the pandemic: changes related to work and finances, changes related to healthcare including access to healthcare, and changes related to aspects of daily life such as childcare, social contact, housing, and recreation (Table 5).

**Table 5:** Summary of themes including a description as well as a respondent’s representative quotation

Theme	Description	Representative Quote
Job loss	Resulted in a reduction or elimination of work	“Stopped working in March and only went back at the beginning of October. Even with unemployment, money has been tight”
Healthcare access/concern	Reduced access to healthcare due to in-person appointment restrictions and/or availability of healthcare staff	“I had limited access to healthcare”
Physical health concern	Increased concern about physical health	“Almost all my appointments are now telehealth, where I have no telehealth appointments prior to COVID-19. My chronic pain treatments were all canceled, as they were in person appointments for injections, medical massages and acupuncture. My health has definitely suffered as a result of COVID-19”
Social / family contact	Reduced contact with friends and family	“Not seeing my family; I isolated”
Financial concern	Stress related to finances due to COVID	“Stress over finances and fear of getting seriously ill”
Mental health concern	Increased mental health concerns during pandemic	“Covid has been the epitome of bad timing, and has affected my mental health most of all. Pain is pain, which is only so manageable, but just when I thought I had a handle on my mental health, Covid came in and reversed so much of my progress”
Remote Work	Resulted in working from home	“Working from home, paranoia about getting sick or bringing the disease home; Limited social activities”
Children's school / care	Reduction or elimination of childcare	“kids are doing a hybrid model at school with remote days and no buses which complicates work and logistics for everyday life”
Weight gain	Weight increases during pandemic	“I have gained some weight (from being less active)”
Reduced recreation	Reduced time spent doing recreational activities during pandemic	“Finances, job, friends, vacation”
Housing	Potential change to or loss of housing	“It is stressful because it may affect my healthcare and housing”
Financial improvement	Finances improved due to receiving unemployment	“Because I got pandemic unemployment for a while, I have more money usual”

### Theme 1: Changes related to work and finances

Emergent themes included reduced or eliminated work, financial concerns, increased working from home, though one person reported that they experienced financial improvement. Respondents generally indicated more financial worry since the beginning of the pandemic. For example, one participant reported:

“Stopped working in March and only went back at the beginning of October. Even with unemployment, money has been tight”.

Other respondents reported concerns related to finances. For example, one respondent reported:

“Stress over finances and fear of getting seriously ill”.

One participant reported an increase in finances due to receiving increased unemployment compensation:

“Because I got pandemic unemployment for a while, I have more money than usual”.

### Theme 2: Health and access to healthcare

Respondents generally highlighted decreased physical and mental health, as well as concerns related to access to healthcare. For example, one respondent reported:

“Limited access to healthcare.”

Another participant commented on mental health:

“Covid has been the epitome of bad timing and has affected my mental health most of all. Pain is pain, which is only so manageable, but just when I thought I had a handle on my mental health, Covid came in and reversed so much of my progress.”

### Theme 3: Life circumstances (childcare, social contact, housing, and reduced recreation)

Some participants focused on changes related to aspects of daily life such as childcare, social contact, housing, and recreation. When asked about how COVID has affected them, one respondent reported:

“Not seeing my family; isolation.”

Another respondent focused on the difficulty with childcare during the pandemic:

“Kids are doing a hybrid model at school with remote days and no buses which complicates work and logistics for everyday life.”

## Discussion

One of the main results of our study was that pain catastrophizing and FM impact increased significantly in women with FM after the pandemic started. Our results further indicate that the current COVID-19 pandemic has had a significant impact of the pandemic on women’s FM symptoms and mental health, including anxiety and depression. Other domains were also adversely affected, with many women reporting a decreased level of activity and a worsening of functioning and sleep quality. Chronic pain is one of the primary causes of disability; it has a significant detrimental impact on other areas of life such as family relations, job loss or job insecurity and general well-being. According to the participant’s responses, the COVID-19 pandemic appears to have substantially affected these areas of life. In addition, the current pandemic seems to have also contributed to challenges in effective pain management of this patient population. Participants reported that they tended to stay away from hospitals both due to the imposed movement limitations, and fear of infection, while others were not able to receive pain treatments due to significant delays. Most did not feel that virtual therapies were sufficient for the treatment of their FM symptoms.

Concerns that FM patients might be extremely vulnerable during COVID-19 have been recently raised by other investigators as well. Mohabbat and colleagues [23] suggest that pandemic-related stressors will likely negatively impact central sensitization which represents a hallmark pain mechanism in FM [24]. Another factor related to increased vulnerability is mood-related symptomatology, which is commonly present in FM. For example, at least one report suggests that the pandemic was a trigger which led to mood dysregulation increasing suicidal ideation as a result [23]. Previous research has identified social isolation as an additional risk factor contributing to physical and mental health deterioration in patients with chronic pain [11, 25, 26]. Therefore, it is not surprising that patients with chronic pain conditions such as FM might be disproportionally affected by social distancing prompted by the current COVID-19 pandemic [27]. As a result of the general worsening of symptoms, greater health care utilization, higher direct and indirect costs (societal and personal) and further loss of productivity are expected [23]. However, access to healthcare is limited in many cases, especially due to cancelled elective procedures and reduced availability of face-to-face contact, requiring a rapid transition to telehealth [27]. Despite some evidence that digital delivery of some interventions (e.g. cognitive behavioral therapy, mindfulness, educational programs, acceptance and commitment therapy) can be as effective for chronic pain patients as face-to-face care [28], there are some challenges and barriers that should be noted. These include low technological literacy and fears of disclosing confidential information through the internet [29]. Other limitations include reduced access to interventions where physical contact is necessary, such as massage therapy, physiotherapy, acupuncture and chiropractic care, among others.

The results of our study support these concerns, as the majority of participants reported an overall worsening of pain, fatigue and other FM symptoms, as well as mental well-being and ability to cope. Moreover, the impact of FM (measured using the FIQR) was found to be significantly higher compared to pre-pandemic levels, similar to previous studies [17]. In contrast, another study assessing the FIQR scores before and during the pandemic (May 2020), did not find significant changes in FIQR. The authors explain this finding by the fact that some participants had the opportunity to introduce beneficial changes in daily and working habits (smart working, regular physical activity) [30].Similarly, Rivera et al. showed that even though 49% of the patients reported symptom worsening, there was no significant change in quality of life due to pandemic-related confinement. The authors attributed the perceived worsening to the patient’s coping styles [31]. Colas et al. found that the pandemic-related lockdown exacerbated symptoms of fibromyalgia, but lifestyle adjustments to fluctuations of these symptoms overall allowed for a better quality of life [32]. This finding might serve as an explanation for the conflicting reports of previous studies regarding FM impact.

Nearly all emergent themes of the questionnaire reflect clearly the stress, isolation, financial pressure and job insecurity the ongoing pandemic has caused for this patient cohort. Our results also noted suboptimal access to health care for the majority of FM patients, and a relatively low utilization of and dissatisfaction related to telehealth. Given that catastrophizing is associated with FM pain and symptom severity (33), it is not surprising that levels of catastrophizing also increased during the pandemic. In fact, our results show that this increase in pain catastrophizing had the strongest association with a worsened perceived ability to cope with pain and a poorer mental health during the pandemic compared to other clinical measures such as changes in pain intensity or FM impact.

This study has limitations that need to be taken into consideration when interpreting its results. First, the study included a relatively small sample size, which potentially reduces generalizability of our results. Second, qualitative data were collected using a semi-structured questionnaire, which was not exhaustive in content, and may have potentially omitted important responses of FM patients to the pandemic. Third, we did not use validated questionnaires to assess anxiety and depression. However, we did include mental health related questions as part of the covid related questions we included in the interviews.

Lastly, participant inclusion in this study required previous participation in the parent study that involved numerous in-person visits, which may be limiting for some more FM patients that have been severely impacted (self-selection bias) or might have limited access to the internet. Notwithstanding its limitations, this mixed methods study provides longitudinal data on important pain-related outcomes which demonstrate a significant increase in FM symptoms including pain catastrophizing during the COVID-19 pandemic. Additionally, it offers insight into patients’ individual experience during the behavioral restrictions imposed by the COVID-19 pandemic. Our results suggest that women with FM might benefit from follow-up visits to assess symptom severity, evaluate psychosocial status (including any changes in pain-related catastrophizing) and re-evaluate treatment regimens. Further research is needed to evaluate the effect of prolonged isolation and other pandemic-related stressors for this patient population.

## Supplementary Information

**Additional file 1**. Qualitative Interview Questions.

## Data Availability

The datasets analyzed during the current study are available from the corresponding author on reasonable request.

## References

[1] Brooks SK, Webster RK, Smith LE, Woodland L, Wessely S, Greenberg N, Rubin GJ (2020). The psychological impact of quarantine and how to reduce it: rapid review of the evidence. Lancet.

[2] Torales J, O'Higgins M, Castaldelli-Maia JM, Ventriglio A (2020). The outbreak of COVID-19 coronavirus and its impact on global mental health. Int J Soc Psychiatry.

[3] Institute of Medicine Committee on Advancing Pain Research C, Education. The National Academies Collection: Reports funded by National Institutes of Health. Relieving Pain in America: A Blueprint for Transforming Prevention, Care, Education, and Research. Washington (DC): National Academies Press (US) National Academy of Sciences.; 2011.

[4] Bennett RM, Jones J, Turk DC, Russell IJ, Matallana L (2007). An internet survey of 2,596 people with fibromyalgia. BMC Musculoskelet Disord.

[5] Fitzcharles MA, Ste-Marie PA, Rampakakis E, Sampalis JS, Shir Y (2016). Disability in fibromyalgia associates with symptom severity and occupation characteristics. J Rheumatol.

[6] Mas AJ, Carmona L, Valverde M, Ribas B (2008). Prevalence and impact of fibromyalgia on function and quality of life in individuals from the general population: results from a nationwide study in Spain. Clin Exp Rheumatol.

[7] Arnold LM, Clauw DJ (2017). Challenges of implementing fibromyalgia treatment guidelines in current clinical practice. Postgrad Med.

[8] Thieme K, Turk DC, Flor H (2004). Comorbid depression and anxiety in fibromyalgia syndrome: relationship to somatic and psychosocial variables. Psychosom Med.

[9] Romeo A, Tesio V, Ghiggia A, Di Tella M, Geminiani GC, Farina B, Castelli L (2022). Traumatic experiences and somatoform dissociation in women with fibromyalgia. Psychol Trauma.

[10] Romeo A, Benfante A, Geminiani GC, Castelli L (2022). Personality, defense mechanisms and psychological distress in women with fibromyalgia. Behav Sci (Basel)..

[11] Nelson S, Cunningham N, Peugh J, Jagpal A, Arnold LM, Lynch-Jordan A, Kashikar-Zuck S (2017). Clinical profiles of young adults with juvenile-onset fibromyalgia with and without a history of trauma. Arthritis Care Res (Hoboken)..

[12] Harris RA (2014). Chronic pain, social withdrawal, and depression. J Pain Res.

[13] Iannuccelli C, Lucchino B, Gioia C, Dolcini G, Favretti M, Franculli D, Di Franco M. Mental health and well-being during the COVID-19 pandemic: stress vulnerability, resilience and mood disturbances in fibromyalgia and rheumatoid arthritis. Clin Exp Rheumatol. 2021;39 Suppl 130(3):153–60 10.55563/clinexprheumatol/4nb0ku 34161226

[14] Cankurtaran D, Tezel N, Ercan B, Yildiz SY, Akyuz EU (2021). The effects of COVID-19 fear and anxiety on symptom severity, sleep quality, and mood in patients with fibromyalgia: a pilot study. Adv Rheumatol..

[15] Elovainio M, Hakulinen C, Pulkki-Råback L, Virtanen M, Josefsson K, Jokela M, Vahtera J, Kivimäki M (2017). Contribution of risk factors to excess mortality in isolated and lonely individuals: an analysis of data from the UK Biobank cohort study. Lancet Public Health..

[16] Matthews T, Danese A, Caspi A, Fisher HL, Goldman-Mellor S, Kepa A, Moffitt TE, Odgers CL, Arseneault L (2019). Lonely young adults in modern Britain: findings from an epidemiological cohort study. Psychol Med.

[17] Salaffi F, Giorgi V, Sirotti S, Bongiovanni S, Farah S, Bazzichi L, Marotto D, Atzeni F, Rizzi M, Batticciotto A, Lombardi G, Galli M, Sarzi-Puttini P (2021). The effect of novel coronavirus disease-2019 (COVID-19) on fibromyalgia syndrome. Clin Exp Rheumatol.

[18] Batres-Marroquín AB, Medina-García AC, Vargas Guerrero A, Barrera-Villalpando MI, Martínez-Lavín M, Martínez-Martínez LA (2020). Effect of COVID-19 pandemic lockdown on fibromyalgia symptoms. J Clin Rheumatol.

[19] Cleeland CS, Ryan KM (1994). Pain assessment: global use of the Brief Pain Inventory. Ann Acad Med Singapore.

[20] Osman A, Barrios FX, Kopper BA, Hauptmann W, Jones J, O'Neill E (1997). Factor structure, reliability, and validity of the Pain Catastrophizing Scale. J Behav Med.

[21] Bennett RM, Friend R, Jones KD, Ward R, Han BK, Ross RL (2009). The Revised Fibromyalgia Impact Questionnaire (FIQR): validation and psychometric properties. Arthritis Res Ther.

[22] Braun V, Clarke V (2006). Using thematic analysis in psychology. Qual Res Psychol.

[23] Ando H, Cousins R, Young C (2014). Achieving Saturation in Thematic Analysis: Development and Refinement of a Codebook. Comprehensive Psychology..

[24] Mohabbat AB, Mohabbat NML, Wight EC. Fibromyalgia and Chronic Fatigue Syndrome in the Age of COVID-19. Mayo Clinic Proceedings: Innovations, Quality & Outcomes. 2020. 10.1016/j.mayocpiqo.2020.08.002 PMC7661943 33204998

[25] Staud R, Rodriguez ME (2006). Mechanisms of disease: pain in fibromyalgia syndrome. Nat Clin Pract Rheumatol.

[26] Sturgeon JA, Dixon EA, Darnall BD, Mackey SC (2015). Contributions of physical function and satisfaction with social roles to emotional distress in chronic pain: a Collaborative Health Outcomes Information Registry (CHOIR) study. Pain.

[27] Karayannis NV, Baumann I, Sturgeon JA, Melloh M, Mackey SC (2019). The impact of social isolation on pain interference: a longitudinal study. Ann Behav Med.

[28] Karos K, McParland JL, Bunzli S, Devan H, Hirsh A, Kapos FP, Keogh E, Moore D, Tracy LM, Ashton-James CE (2020). The social threats of COVID-19 for people with chronic pain. Pain.

[29] Martorella G, Boitor M, Berube M, Fredericks S, Le May S, Gélinas C (2017). Tailored Web-Based Interventions for Pain: Systematic Review and Meta-Analysis. J Med Internet Res.

[30] Parker S, Prince A, Thomas L, Song H, Milosevic D, Harris MF (2018). Electronic, mobile and telehealth tools for vulnerable patients with chronic disease: a systematic review and realist synthesis. BMJ Open.

[31] Cavalli G, Cariddi A, Ferrari J, Suzzi B, Tomelleri A, Campochiaro C, De Luca G, Baldissera E, Dagna L (2021). Living with fibromyalgia during the COVID-19 pandemic: mixed effects of prolonged lockdown on the well-being of patients. Rheumatology (Oxford).

[32] Rivera J, Castrejón I, Vallejo-Slocker L, Offenbächer M, Molina-Collada J, Trives L, López K, Caballero L, Hirsch JK, Toussaint L, Nieto JC, Alvaro-Gracia JM, Vallejo MA (2021). Clinical impact of confinement due to the COVID-19 pandemic on patients with fibromyalgia: a cohort study. Clin Exp Rheumatol.

[33] Colas C, Jumel A, Vericel M-P, Barth N, Manzanares J, Goutte J, Fontana L, Féasson L, Hupin D, Guyot J (2021). Understanding Experiences of Fibromyalgia Patients Involved in the Fimouv Study During COVID-19 Lockdown. Front Psychol.

[34] Carriere JS, Lazaridou A, Martel MO, Cornelius M, Campbell C, Smith M, Haythornthwaite JA, Edwards RR (2019). The moderating role of pain catastrophizing on the relationship between partner support and pain intensity: a daily diary study in patients with knee osteoarthritis. J Behav Med.

