# Supplementary material for: The impact of COVID-19 pandemic on mental and physical wellbeing in women with fibromyalgia: a longitudinal mixed-methods study
Source: BMC Womens Health. 2022 Jun 30;22:267. doi: 10.1186/s12905-022-01840-9 (PMC9245870; doi:10.1186/s12905-022-01840-9)
Supplement: Supplementary file 1 — Additional file 1. Qualitative Interview Questions. [file 12905_2022_1840_MOESM1_ESM.docx]

A Qualitative Study on the Psychological Experience of Fibromyalgia Patients during the COVID-19 Pandemic

1. Has the pandemic impacted your life? Which changes has the pandemic brought in your work and life? (e.g. health, family members, job, finances, access to healthcare)
2. What response best describes how the **COVID-19 pandemic** has impacted your life overall?
3. Very positively overall
4. Somewhat positively overall
5. A little bit positively overall
6. Positive and negative impacts equally balanced overall
7. A little bit negatively overall
8. Somewhat negatively overall
9. Very negatively overall
10. What response best describes your personal experience with **the COVID-19 pandemic**?
    1. I was diagnosed with COVID-19 by a medical professional which was confirmed with a laboratory test.
    2. I developed symptoms consistent with COVID-19, but was never tested.
    3. I don’t think I ever had COVID-19.
    4. Other (please specify):________________________________________

Were you hospitalized (or did you have to stay in the hospital overnight) while you had the coronavirus? Yes No

1. Has anyone you live with had **COVID-19**?
   1. Someone I live with was diagnosed with COVID-19 by a medical professional which was confirmed with a laboratory test.
   2. Someone I live with developed symptoms consistent with COVID-19, but was never tested.
   3. I don’t think anyone I live with ever had COVID-19.
   4. Other (please specify):________________________________________

Was anyone you live with hospitalized (or did they have to stay in the hospital overnight) while they had the coronavirus? Yes No

1. What response best describes how **the COVID-19 pandemic** has impacted your access to healthcare (including medications, healthcare visits, other treatments)?
2. Very much improved
3. Much improved
4. Minimally improved
5. No change
6. Minimally worse
7. Much worse
8. Very much worse
9. Did you receive fibromyalgia-related medical advice/ treatments via telehealth? Yes No
10. Do you feel virtual therapies provided as an alternative has been sufficient for your pain-related needs? Yes No
11. What response best describes how **the COVID-19 pandemic** has impacted your ability to meet your basic needs (including housing, food, essential supplies, etc.)?
12. Very much improved
13. Much improved
14. Minimally improved
15. No change
16. Minimally worse
17. Much worse
18. Very much worse
19. What response best describes how **the COVID-19 pandemic** has influenced your pain on average in the past week?
    - Very much improved
    - Much improved
    - Minimally improved
    - No change
    - Minimally worse
    - Much worse
    - Very much worse
20. What response best describes how **the COVID-19 pandemic** has impacted your ability to cope with your pain during the past week?
21. Very much improved
22. Much improved
23. Minimally improved
24. No change
25. Minimally worse
26. Much worse
27. Very much worse
28. What response best describes how **the COVID-19 pandemic** has impacted your use of pain medication during the past week?
29. Very much increased
30. Much increased
31. Minimally increased
32. No change
33. Minimally decreased
34. Much decreased
35. Very much decreased
36. Please tell us about the impact of the pandemic on your fibromyalgia symptoms (other than pain).
    - Have your pain levels increased?
    - Has your fatigue increased?
    - Did any other symptoms worsen?
    - What do you think is the reason for your symptoms worsening?
37. What response best describes how **the COVID-19 pandemic** has influenced your enjoyment of life in the past week?
    - Very much improved
    - Much improved
    - Minimally improved
    - No change
    - Minimally worse
    - Much worse
    - Very much worse
38. What response best describes how **the COVID-19 pandemic** has impacted your mental health (including anxiety, depression, stress, mood)?
39. Very much improved
40. Much improved
41. Minimally improved
42. No change
43. Minimally worse
44. Much worse
45. Very much worse
46. Please tell us how the pandemic influences your mental well-being.
    - Have you been more anxious than usual?
    - Have you had trouble sleeping?
    - Have you been experiencing low mood?
47. What response best describes how **the COVID-19 pandemic** has influenced your general activity in the past week?
    - Very much improved
    - Much improved
    - Minimally improved
    - No change
    - Minimally worse
    - Much worse
    - Very much worse
48. What coping strategies have you been using to make daily life easier while staying at home/working from home /resuming your work responsibilities during the pandemic?
49. How optimistic are you about the outcome of the pandemic and returning to “normal” life?
